# Designing a new integrated control solution for electric power steering systems based on a combination of nonlinear techniques

**DOI:** 10.1371/journal.pone.0308530

**Published:** 2024-09-16

**Authors:** Tuan Anh Nguyen

**Affiliations:** Thuyloi University, Hanoi, Vietnam; Buckinghamshire New University - High Wycombe Campus: Buckinghamshire New University, UNITED KINGDOM OF GREAT BRITAIN AND NORTHERN IRELAND

## Abstract

An EPS system is used to improve the stability and safety of the car when steering while also simplifying the steering process. This article introduces a novel control solution for the EPS system called BSSMCPID. This algorithm combines two nonlinear techniques, BS and SMC, with the input signal corrected by a PID technique. This algorithm provides three new contributions compared to existing algorithms: reducing system errors and eliminating phase differences, ensuring stability even when exposed to external disturbances, and reducing power consumption. The system’s stability is evaluated according to the Lyapunov criterion, while the algorithm’s performance is evaluated based on numerical simulation results. According to the article findings, the RMS error of the steering column angle and steering motor angle values (controlled objects) is approximately zero, and the RMS error of the steering column speed and steering motor speed is about 0.01 rad/s, which is much lower than the results obtained with traditional BS and PID controllers. When the EPS system is controlled by the integrated nonlinear method proposed in this work, the output values always closely follow the reference values with negligible errors under all investigated conditions. Additionally, power steering performance increases as speed decreases or driver torque increases, which follows the ideal assisted power steering curve. In general, the responsiveness and stability of the system are always ensured when applying this algorithm.

## Introduction

The electric power steering system is commonly equipped on many modern vehicles today. This system reduces the impact force on the steering wheel and enhances the steering feel at different speeds [1]. Compared to HPS and EHPS systems, the energy consumption of EPS was lower, according to Baharom et al. [2]. Besides, its structure was less complicated than other types and easy to arrange on many car models. According to Ramasamy, the EPS system positively impacted vehicle handling, while steering feel and comfort were always guaranteed [3]. In [4], Park et al. showed that energy consumption efficiency could be improved using EPS systems instead of conventional HPS. Using traditional HPS or EHPS systems often causes vehicle vibration and noise [5]. The main reason for this was the continuous operation of hydraulic pumps, which operated even without steering [6]. Replacing traditional HPS and EHPS systems with EPS could help improve this. According to Khalkhali et al., electric power steering systems have been applied to many vehicles, such as sedans, hatchbacks, CUVs, SUVs, pickups, and minivans [7]. The electric motor of the EPS system was mounted on the steering column for these vehicles (C-EPS). The EPS system should be rack-mounted for heavy trucks, which is called R-EPS, according to Jang et al. [8]. Ramasamy pointed out that the C-EPS system used a simple DC motor, while the R-EPS was equipped with a BLDC motor, belt, many balls, and other components. Therefore, the cost of R-EPS was higher than C-EPS [3].

**Fig 1:**
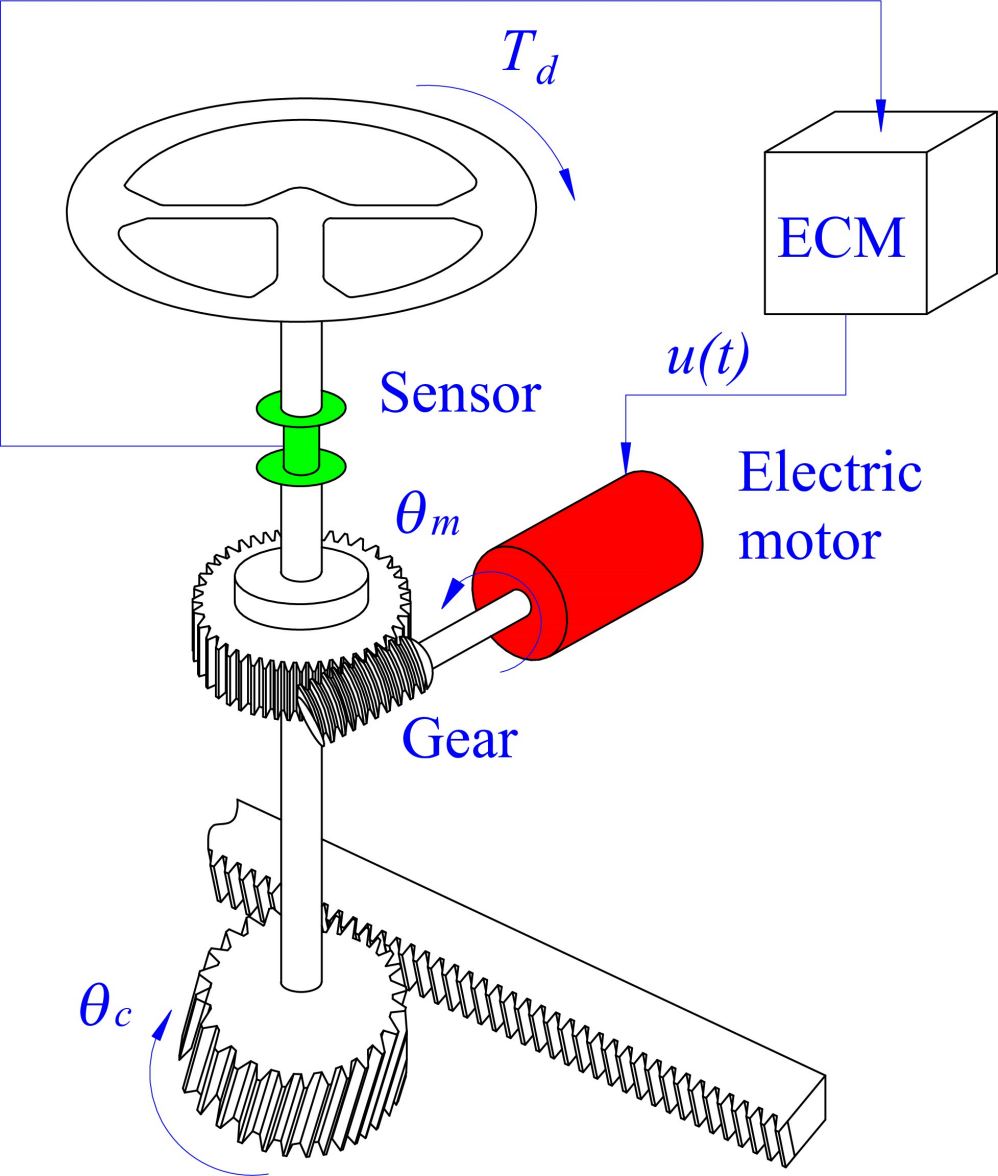
EPS system structural.

**Fig 2:**
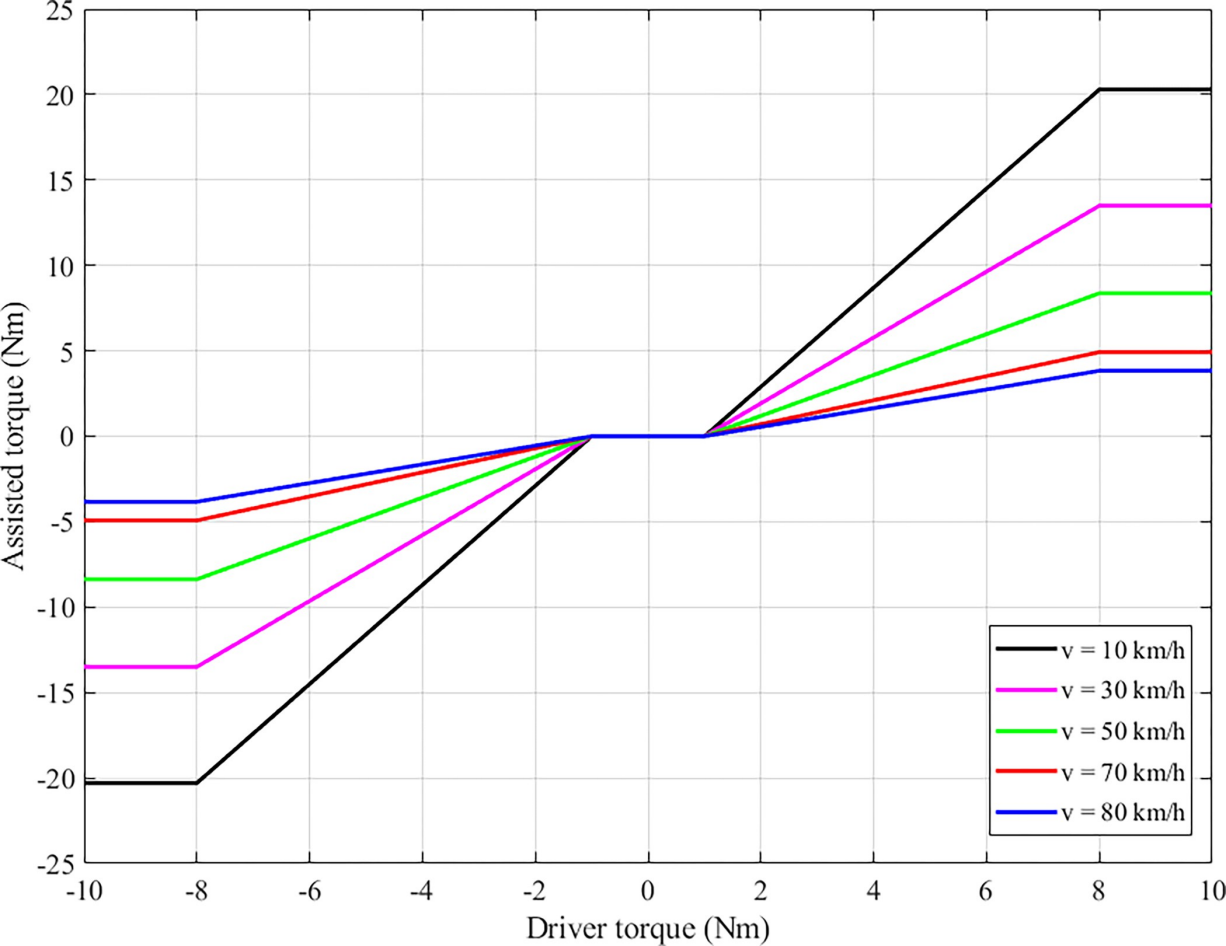
Ideal assisted power steering curve.

The typical structure of a C-EPS system is illustrated in Fig 1, including an electric motor, a pair of gears mounted on the motor’s output shaft, Hall or photoelectric sensors, an ECM, a steering wheel, a pinion, and a rack. Fig 2 depicts an EPS system’s ideal assisted power steering curves. These curves are established based on experimental values. According to this description, assisted torque (after being amplified by the gear pair) will be zero if |*T*_*d*_| < |*T*_*dmin*_|. This value will increase linearly as |*T*_*dmin*_| ≤ |*T*_*d*_| ≤ |*T*_*dmax*_|, becoming saturated (*T*_*a*_ = *T*_*amax*_) when *T*_*d*_ exceeds its limit value (*T*_*dmax*_). In addition, the assisted torque value depends on the car’s speed. Power steering performance is highest when the automobile steers at shallow speed. On the contrary, this value can decrease sharply as the speed increases. This helps improve the steering experience and safety for users. The relationship between assisted torque, velocity, and driver torque is shown in Eq (1). The coefficients *ξ*_i_ in Eq (2) should be referred to in ref [9]. Some 3D characteristic curves, which consider the influence of many other factors, were presented in refs [10, 11].


$$T_{a}=\{\begin{array}{l} 0\;0\leq T_{d}<T_{dmin}\\ f(T_{d},v)\;T_{dmin}\leq T_{d}\leq T_{dmax}\\ T_{amax}\;T_{d}>T_{dmax} \end{array}$$ (1)

$$f(T_{d},v)=(\xi_{1}v^{2}+\xi_{2}v+\xi_{3})(T_{d}-T_{dmin})$$ (2)

Road reaction torque (*T*_*r*_) is an essential component that must be determined before studying the EPS control system. Some previous publications often omitted to calculate this value. Instead, researchers assumed this was an external disturbance and knew its value in advance [12]. In ref [13], Jang et al. presented a simple method to determine *T*_*r*_. In ref [14], Marouf et al. used a linear single-track dynamics model to determine the change in road reaction torque. This is a feasible solution and has been applied in several other studies. In general, determining the change in Tr is extremely important, and we should calculate this value accurately instead of assuming it is an external disturbance.

Compared with other mechatronic systems in cars, such as automatic braking systems [15], active suspension systems [16], or active anti-roll systems [17], the number of studies on the EPS system is less. Research on control for steering systems published in recent years could be divided into two main categories: linear control and nonlinear control. Several traditional PID algorithms regarding linear control have been widely applied to the EPS system. In ref [18], Guan et al. applied the PID technique to determine reference steering motion intensity. The classic PID controller for the EPS system was shown in ref [19] by Hassan et al. The parameters of this controller were optimally calculated based on a BCGA so that the value of MSE converges to zero [19]. However, this calculation process was only presented in a very brief and straightforward way and lacked evaluation. The idea of using the PSO technique to optimize PID controller parameters was proposed by Hanifah et al. in ref [20]. However, the difference between the conventional PID controller and the PID-PSO-tuned controller was slight [20]. The calculation results in ref [21] showed that the average power was reduced by 0.82% when using the PSO technique to tune the parameters for the PID controller, while the ACO technique could help reduce it by 7.41%. In ref [22], Li et al. applied the BPNN technique to determine ideal values for the PID controller. The structure of this learning algorithm included three layers; the inputs were vehicle speed and driver torque, while the output was the desired current. Compared with traditional PID controllers, the output response signal, when optimally calculated, was quieter and smoother. However, phase lag occurred when steering at low speed [22]. A linear filter could improve the phase delay problem but cause its error to increase [23]. In ref [24], Zheng and Wei designed a phase-compensated fuzzy PI controller for the EPS system. Simulation results from ref [24] illustrated that the output signal closely matches the desired signal. However, this was only true under certain conditions, and the influence of interference must be completely eliminated. Some EPS systems for large vehicles use two motors controlled by two independent PID controllers [25, 26]. When considering the influence of external disturbances, the output signals were chattered [27, 28], even leading to a phase difference [28]. Crosswinds, road surface irregularities, and other factors were all considered external disturbances [29]. The PID technology was only applied to single-input and single-output systems. The solution using the LQR technique was highly feasible when the system had many inputs and many outputs. In ref [30], Chitu et al. introduced a digital LQR technique for automotive EPS systems. This technique aimed to minimize a cost function [30]. An LQG controller was developed by combining LQR and LQE techniques, according to Irmer and Henrichfreise [31]. The controller parameters were optimally calculated by GA, but the error between the results was not large [32]. Equipping a Kalman filter could help the system partially limit the influence of external disturbances [33]. Several other linear control techniques for EPS systems were also mentioned in refs [34, 35].

The linear control techniques mentioned above are simple and commonly applied to many mechatronic systems today. However, the controller’s performance may be degraded if the system is nonlinear or subject to external nonlinear disturbances. In ref [36], Lee et al. showed that the output signal obtained from a simple linear controller did not converge. The simulation results in ref [37] illustrated that the step response of H_∞_ control was more efficient than that of conventional PID. According to Zhao and Zhang [38], an overshoot occurred when applying the PID technique compared to H_2_/ H_∞_. In another comparison, Dannöhl et al. showed that the response of nonlinear controllers was more efficient than that of conventional linear controllers [39]. In general, nonlinear control techniques often provide superior system performance compared to classical linear control techniques.

SMC is a well-known control technique applied to nonlinear systems [40]. In ref [41], Lee et al. applied the adaptive SMC technique to the EPS system. The controlled object in ref [41] was the steering wheel angle, and the inputs were driver torque and vehicle speed. The calculation results showed that the controlled object followed the desired value. However, the chattering phenomenon occurred strongly for other state variables (steering wheel speed and acceleration). In ref [42], Kim et al. proposed a robust SMC algorithm to control steering wheel torque (controlled object). An extended state observer was integrated with this controller to limit the influence of external disturbances, according to Kim et al. The value of output torque followed the desired value with a small error. However, chattering still occurred under computational conditions, negatively affecting system quality [42]. The calculation results in ref [43] also showed that chattering and phase difference phenomena still occurred when applying the SMC technique. This was a characteristic phenomenon of the SMC algorithm, and it was not easy to eliminate, according to Nguyen and Nguyen [44]. An integrated controller between SMC and PID was designed in ref [45] to solve this problem (the output of the SMC controller was the reference signal of the PID controller). The output signals became smoother when combining the SMC technique with adaptive fuzzy algorithms, and the chattering phenomenon could be reduced [46, 47]. However, this phenomenon could only be partially solved if the system was improved with complex, intelligent algorithms that combine fuzzy techniques and neural networks [48]. In ref [49], Na et al. applied another nonlinear technique to the EPS system for rejecting disturbances, called ADRC. The object controlled in ref [49] was the steering wheel torque. Calculation results showed that chattering phenomena still occurred strongly for uncontrolled state variables (steering angle and steering speed). When applying the ADRC technique to the EPS system, the results obtained from the controlled and uncontrolled scenarios were similar, with negligible differences [50]. Fu et al. showed another nonlinear control technique for torque oscillation suppression in electric steering systems in ref [51]. However, this could not eliminate the chattering problem. This phenomenon remained strong even when applying the MPC technique, as shown in ref [52]. Applying BS nonlinear control techniques to the automotive steering system is a highly effective solution. This well-known technique has been applied to many industrial systems, but it has only recently been applied to power steering systems. In ref [53], Shi et al. designed a BS controller for the EHPS system to control steering wheel angle. The calculation results in ref [53] illustrated that the error of the BS algorithm was smaller than that of ADRC and fuzzy PI, and the chattering phenomenon almost did not occur. However, the system’s stability would be strongly reduced once the input signal had too high a frequency, according to Shi et al. [53]. In ref [54], Nguyen and Nguyen designed a BSPID algorithm to improve the performance of the EPS system. The final control signal was synthesized from two component signals, PID and BS, and they were amplified with optimal values, which were determined through a loop algorithm. The graphs in ref [54] illustrated that the simulation results obtained from the BSPID algorithm tended to follow the reference value with negligible error. The system’s stability was proven based on Lyapunov’s theorem for the BS technique, while the PID technique was not mentioned. Therefore, the performance of the BSPID algorithm could only be correct under the optimal conditions mentioned in ref [54]. To improve this problem, another integrated controller was introduced in a recent publication by Nguyen [55]. This controller was a combination of BS and PID techniques with flexibly adjusted parameters based on fuzzy set theory. In addition, the input of the BS technique was the output of the PID technique. Therefore, the system’s stability could be fully proven according to the Lyapunov criterion. The results from ref [55] showed that the system error was negligible, and the chattering phenomenon was completely eliminated. However, this algorithm only had a single controlled object (steering motor angle). Therefore, the errors of other control variables might not be guaranteed under severe motion conditions (deficient speed, ample driver torque, and frequency).

Although the above algorithms can bring high performance to the EPS system, some drawbacks still exist. Firstly, linear control techniques (such as LQR, PID, or LPV) cannot guarantee system performance under harsh working conditions, causing system errors to increase. In addition, external disturbances will significantly affect the system if it is controlled by classical linear techniques. Besides, phase differences and signal noise still exist. Secondly, nonlinear control techniques (such as SMC or ADRC) can reduce system errors but cause chattering, negatively affecting system stability. When using two nonlinear techniques to control two different state variables, the control model will become highly complicated, while the chattering phenomenon may occur more strongly. Fuzzy control methods can be integrated with nonlinear techniques to reduce chattering, but it requires much experience from the researcher when designing membership functions and fuzzy rules. Thirdly, existing BS techniques (where only one object is controlled) cannot meet the requirements for system stability when steering in extreme conditions. Fourthly, road reaction torque should be determined using a dynamic model instead of assuming this is known in advance. Finally, the influence of other disturbances also needs to be considered when investigating the system.

This article proposes a nonlinear integrated algorithm to address the above drawbacks. This algorithm is a combination of techniques: SMC, BS, and PID, so it is called BSSMCPID. The SMC technique is applied to control the first object (steering column angle), and the BS technique is applied to control the second object (steering motor angle), while the input of the nonlinear control technique is adjusted through the PID method. This combination provides two outstanding contributions: 1) Controlling multi-input and multi-output systems to reduce systematic errors, while previous studies only controlled a single object; 2) The proposed algorithm is formed based on the combination of many different control techniques to reduce the influence of chattering (compared to conventional SMC) and eliminate phase delay phenomena (compared to traditional BS and classic PID). Moreover, we consider the impact of additional disturbances when examining steering behavior, rather than presuming it is known beforehand. These are the novel contributions to the article, which differ from previous publications.

The structure of this article includes 4 sections: the introduction, the material and method, the result and discussion, and the conclusion. The mathematical model and control algorithms will be presented in the article’s next section.

## 2. Material and method

### 2.1. Vehicle and EPS models

The dynamics of the C-EPS system (Fig 1) are described by Eqs (3)–(5).

$$J_{c}{\ddot{\theta }}_{c}+B_{c}{\dot{\theta }}_{c}+K_{c}\theta_{c}=\frac{K_{c}}{N}\theta_{m}+T_{d}$$ (3)

$$\frac{K_{c}}{N}\theta_{c}+K_{t}i_{m}-\frac{T_{r}}{N}=J_{eq}{\ddot{\theta }}_{m}+B_{eq}{\dot{\theta }}_{m}+\frac{K_{c}+K_{r}r_{p}^{2}}{N^{2}}\theta_{m}$$ (4)

$$K_{t}{\dot{\theta }}_{m}+L_{m}{\dot{i}}_{m}+R_{m}i_{m}=u(t)$$ (5)

where

$$B_{eq}=B_{m}+\frac{r_{p}^{2}}{N^{2}}B_{r}$$ (6)

$$J_{eq}=J_{m}+\frac{r_{p}^{2}}{N^{2}}M_{r}$$ (7)

Road reaction torque (*T*_*r*_) is calculated according to Eq (8), and external disturbance torque (*T*_*ed*_) is illustrated in Fig 5B.


$$T_{r}\approx r_{p}l_{c}\frac{cos^{2}(\gamma_{k})cos^{2}(\gamma_{c})}{l_{n}}F_{y1}+T_{ed}$$ (8)

The tire is assumed to be deformed in the linear region (steering angle and vehicle speed are small). Then, the lateral force at the front and rear tires are calculated according to Eqs (9) and (10), respectively.


$$F_{y1}=-C_{\alpha 1}\alpha_{1}$$ (9)

$$F_{y2}=-C_{\alpha 2}\alpha_{2}$$ (10)

**Fig 3:**
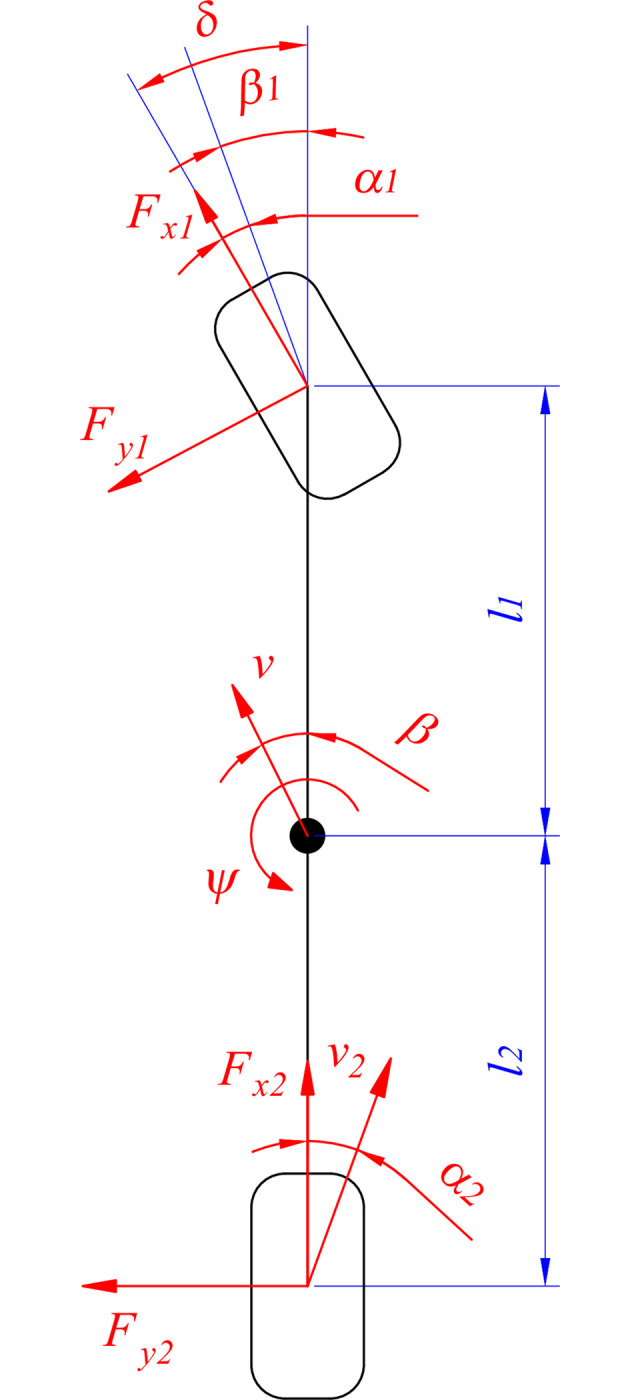
Linear single-track dynamics model.

In this work, we establish a linear single-track dynamics model to determine the motion of the car (Fig 3). This model calculates the tire slip angle *α*_*i*_ according to (11) and (12).


$$\alpha_{1}=\frac{v_{y}+l_{1}\dot{\psi }}{v_{x}}-\delta$$ (11)

$$\alpha_{2}=\frac{v_{y}-l_{2}\dot{\psi }}{v_{x}}$$ (12)

The steering angle (*δ*) is determined through driver torque (*T*_*d*_), while longitudinal velocity (*v*_*x*_), lateral velocity (*v*_*y*_), and yaw angle (*ψ*) are calculated according to the dynamic model. These are described in Eqs (13)–(15).


$$m({\dot{v}}_{x}-\dot{\psi }v_{y})=\sum_{i=1}^{2}F_{xi}$$ (13)

$$m({\dot{v}}_{y}+\dot{\psi }v_{x})=\sum_{i=1}^{2}F_{yi}$$ (14)

$$J_{\psi }\ddot{\psi }=\sum_{i=1}^{2}{\left(-1\right)}^{i-1}l_{i}F_{yi}$$ (15)

The automotive dynamics model and the EPS system model can be considered linear. However, the effects of external disturbances (input excitations) are nonlinear. Therefore, applying robust nonlinear control techniques is expected to provide greater efficiency in controlling system performance.

### 2.2. Control model

In this work, two objects need to be controlled (the steering column angle and the steering motor angle). The steering column angle is controlled by the SMC algorithm, while the other is controlled by the BS algorithm.

Set the state variables as follows:

$$x_{1}=\theta_{c}$$ (16)

$$x_{2}={\dot{\theta }}_{c}$$ (17)

$$x_{3}=\theta_{m}$$ (18)

$$x_{4}={\dot{\theta }}_{m}$$ (19)

$$x_{5}=i_{m}$$ (20)

Differentiating the state variables from (16) to (20), we obtain Eqs (21)–(25), respectively.


$${\dot{x}}_{1}=x_{2}$$ (21)

$${\dot{x}}_{2}=-\frac{K_{c}}{J_{c}}x_{1}-\frac{B_{c}}{J_{c}}x_{2}+\frac{K_{c}}{J_{c}N}x_{3}+\frac{T_{d}}{J_{c}}$$ (22)

$${\dot{x}}_{3}=x_{4}$$ (23)

$${\dot{x}}_{4}=\frac{K_{c}}{J_{eq}N}x_{1}-\frac{K_{c}+K_{r}r_{p}^{2}}{J_{eq}N^{2}}x_{3}-\frac{B_{eq}}{J_{eq}}x_{4}+\frac{K_{t}}{J_{eq}}x_{5}-\frac{T_{r}}{J_{eq}N}$$ (24)

$${\dot{x}}_{5}=-\frac{K_{t}}{L_{m}}x_{4}-\frac{R_{m}}{L_{m}}x_{5}+\frac{1}{L_{m}}u(t)$$ (25)

Eqs (26)–(29) correspond to the second, third, fourth, and fifth derivatives of the first control variable (*x*_1_), respectively.

$${\ddot{x}}_{1}={\dot{x}}_{2}=-\frac{K_{c}}{J_{c}}x_{1}-\frac{B_{c}}{J_{c}}x_{2}+\frac{K_{c}}{J_{c}N}x_{3}+\frac{T_{d}}{J_{c}}$$ (26)

$${\dddot{x}}_{1}=\sum_{i=1}^{4}a_{i}x_{i}+(-\frac{B_{c}T_{d}}{J_{c}^{2}}+\frac{{\dot{T}}_{d}}{J_{c}})$$ (27)

$$x_{1}^{\left(4\right)}=\sum_{i=1}^{5}b_{i}x_{i}+(\frac{a_{2}T_{d}}{J_{c}}-\frac{B_{c}{\dot{T}}_{d}}{J_{c}^{2}}+\frac{{\ddot{T}}_{d}}{J_{c}})-\frac{a_{4}T_{r}}{J_{eq}N}$$ (28)

$$\begin{array}{l} x_{1}^{\left(5\right)}=\sum_{i=1}^{5}c_{i}x_{i}+(\frac{b_{2}T_{d}}{J_{c}}+\frac{a_{2}{\dot{T}}_{d}}{J_{c}}-\frac{B_{c}{\ddot{T}}_{d}}{J_{c}^{2}}+\frac{{\dddot{T}}_{d}}{J_{c}})+(-\frac{b_{4}T_{r}}{J_{eq}N}-\frac{a_{4}{\dot{T}}_{r}}{J_{eq}N})+\frac{b_{5}u(t)}{L_{m}}\\ =f(x)+f(T_{d})+f(T_{r})+f(u(t)) \end{array}$$ (29)

where

$$a_{1}=\frac{B_{c}K_{c}}{J_{c}^{2}}\;b_{1}=\frac{a_{4}K_{c}}{J_{eq}N}-\frac{a_{2}K_{c}}{J_{c}}\;c_{1}=\frac{b_{4}K_{c}}{J_{eq}N}-\frac{b_{2}K_{c}}{J_{c}}$$

$$a_{2}=-\frac{K_{c}}{J_{c}}+\frac{B_{c}^{2}}{J_{c}^{2}}\;b_{2}=a_{1}-\frac{a_{2}B_{c}}{J_{c}}\;c_{2}=b_{1}-\frac{b_{2}B_{c}}{J_{c}}$$

$$a_{3}=-\frac{B_{c}K_{c}}{J_{c}^{2}N}\;b_{3}=\frac{a_{2}K_{c}}{J_{c}N}-\frac{a_{4}(K_{c}+K_{r}r_{p}^{2})}{J_{eq}N^{2}}\;c_{3}=\frac{b_{2}K_{c}}{J_{c}N}-\frac{b_{4}(K_{c}+K_{r}r_{p}^{2})}{J_{eq}N^{2}}$$

$$a_{4}=\frac{K_{c}}{J_{c}N}\;b_{4}=a_{3}-\frac{a_{4}B_{eq}}{J_{eq}}\;c_{4}=b_{3}-\frac{b_{4}B_{eq}}{J_{eq}}-\frac{b_{5}K_{t}}{L_{m}}$$

$$b_{5}=\frac{a_{4}K_{t}}{J_{eq}}\;c_{5}=\frac{b_{4}K_{t}}{J_{eq}}-\frac{b_{5}R_{m}}{L_{m}}$$ (30)

The first error of the system (*e*_1_) is given as (31) with *x*_1*ref*_ = *x*_1*ideal*_.


$$e_{1}=x_{1ref}-x_{1}$$ (31)

Taking the derivative of the error *e*_1_, we get Eqs from (32) to (36).


$${\dot{e}}_{1}={\dot{x}}_{1ref}-x_{2}$$ (32)

$${\ddot{e}}_{1}={\ddot{x}}_{1ref}-(-\frac{K_{c}}{J_{c}}x_{1}-\frac{B_{c}}{J_{c}}x_{2}+\frac{K_{c}}{J_{c}N}x_{3}+\frac{T_{d}}{J_{c}})$$ (33)

$${\dddot{e}}_{1}={\dddot{x}}_{1ref}-[\sum_{i=1}^{4}a_{i}x_{i}+(-\frac{B_{c}T_{d}}{J_{c}^{2}}+\frac{{\dot{T}}_{d}}{J_{c}})]$$ (34)

$$e_{1}^{\left(4\right)}=x_{1ref}^{\left(4\right)}-[\sum_{i=1}^{5}b_{i}x_{i}+(\frac{a_{2}T_{d}}{J_{c}}-\frac{B_{c}{\dot{T}}_{d}}{J_{c}^{2}}+\frac{{\ddot{T}}_{d}}{J_{c}})-\frac{a_{4}T_{r}}{J_{eq}N}]$$ (35)

$$e_{1}^{\left(5\right)}=x_{1ref}^{\left(5\right)}-x_{1}^{\left(5\right)}$$ (36)

The sliding surface (*σ*) is an essential component of the SMC algorithm. A linear sliding surface is selected as (37); *k*_*i*_ are the coefficients of the polynomial chosen so that (38) satisfies the Hurwitz condition.


$$\sigma =e_{1}^{\left(4\right)}+k_{1}{\dddot{e}}_{1}+k_{2}{\ddot{e}}_{1}+k_{3}{\dot{e}}_{1}+k_{4}e_{1}$$ (37)

$$\Omega (s)=s^{4}+k_{1}s^{3}+k_{2}s^{2}+k_{3}s+k_{4}$$ (38)

A Lyapunov function is chosen as (39) such that it is positive definite ∀*x* ≠ 0. The Eq (40) is obtained by taking the derivative of *V*_1_(*x*).


$$V_{1}(x)=\frac{1}{2}\sigma^{2}$$ (39)

$${\dot{V}}_{1}(x)=\sigma \dot{\sigma }$$ (40)

Taking the derivative of Eq (37), we get (41). Combining (36) and (41), we obtain (42).


$$\dot{\sigma }=e_{1}^{\left(5\right)}+k_{1}e_{1}^{\left(4\right)}+k_{2}{\dddot{e}}_{1}+k_{3}{\ddot{e}}_{1}+k_{4}{\dot{e}}_{1}$$ (41)

$$\dot{\sigma }=x_{1ref}^{\left(5\right)}-x_{1}^{\left(5\right)}+k_{1}e_{1}^{\left(4\right)}+k_{2}{\dddot{e}}_{1}+k_{3}{\ddot{e}}_{1}+k_{4}{\dot{e}}_{1}$$ (42)

The Eq (43) is obtained by substituting Eq (29) into (42).


$$\dot{\sigma }=x_{1ref}^{\left(5\right)}-[f(x)+f(T_{d})+f(T_{r})+f(u_{1}(t))]+k_{1}e_{1}^{\left(4\right)}+k_{2}{\dddot{e}}_{1}+k_{3}{\ddot{e}}_{1}+k_{4}{\dot{e}}_{1}$$ (43)

The sliding surface (*σ*) is chosen to satisfy condition (44) so that *K* is a positive constant.


$$\dot{\sigma }=-Ksat(\sigma )$$ (44)

The control signal *u*_1_(*t*) is obtained by substituting Eq (44) into Eq (43), as shown in Eq (45).


$$u_{1}(t)=\frac{L_{m}}{b_{5}}[-f(x)-f(T_{d})-f(T_{r})+\sum_{i=1}^{4}k_{i}e_{1}^{\left(5-i\right)}+x_{1ref}^{\left(5\right)}+Ksat(e_{1}^{\left(4\right)}+\sum_{i=1}^{4}k_{i}e_{1}^{\left(4-i\right)})]$$ (45)

The steering motor angle (*x*_3_) is controlled by the BSPID technique. Let *e*_2_ be the error between the second controlled and reference signals.


$$e_{2}=x_{3}-x_{3ref}$$ (46)

The Eq (47) is obtained by taking the derivative of the second error (*e*_2_), which is described in (46).


$${\dot{e}}_{2}=x_{4}-{\dot{x}}_{3ref}$$ (47)

Let *e*_3_ be the first virtual error of the system and *e*_4_ be the second virtual error, with *λ*_1_ and *λ*_2_ being virtual control variables. The first virtual control variable is selected according to Eq (50). This choice satisfies Eq (51).


$$e_{3}=x_{4}-\lambda_{1}$$ (48)

$$e_{4}=x_{5}-\lambda_{2}$$ (49)

$$\lambda_{1}=-d_{1}e_{2}+{\dot{x}}_{3ref}$$ (50)

$$e_{3}=x_{4}-(-d_{1}e_{2}+{\dot{x}}_{3ref})={\dot{e}}_{2}+d_{1}e_{2}\overset{e_{2}\to 0}{\to }{\dot{e}}_{2}$$ (51)

The Eq (52) is obtained by combining Eqs (47), (48), and (50).


$${\dot{e}}_{2}=e_{3}-d_{1}e_{2}$$ (52)

Taking the derivative of Eqs (48) and (50), we get (53) and (54), respectively.


$${\dot{e}}_{3}={\dot{x}}_{4}-{\dot{\lambda }}_{1}$$ (53)

$${\dot{\lambda }}_{1}=-d_{1}{\dot{e}}_{2}+{\ddot{x}}_{3ref}$$ (54)

The Eq (55) is established based on the combination of Eqs (52)–(54).


$${\dot{e}}_{3}={\dot{x}}_{4}+d_{1}(e_{3}-d_{1}e_{2})-{\ddot{x}}_{3ref}$$ (55)

Substituting Eq (24) into (55), we get (56) as below.


$${\dot{e}}_{3}=\frac{K_{c}}{J_{eq}N}x_{1}-\frac{K_{c}+K_{r}r_{p}^{2}}{J_{eq}N^{2}}x_{3}-\frac{B_{eq}}{J_{eq}}x_{4}+\frac{K_{t}}{J_{eq}}x_{5}-\frac{T_{r}}{J_{eq}N}+d_{1}e_{3}-d_{1}^{2}e_{2}-{\ddot{x}}_{3ref}$$ (56)

The coefficient *d*_2_ is selected according to (57). The Eq (58) is obtained by substituting Eqs (48) and (50) into (56).


$$d_{2}=\frac{B_{eq}}{J_{eq}}-d_{1}$$ (57)

$$\begin{array}{l} {\dot{e}}_{3}=\frac{K_{c}}{J_{eq}N}x_{1}+\left(d_{1}\frac{B_{eq}}{J_{eq}}-\frac{K_{c}+K_{r}r_{p}^{2}}{J_{eq}N^{2}}\right)x_{3}+\frac{K_{t}}{J_{eq}}x_{5}\\ -\left(d_{1}\frac{B_{eq}}{J_{eq}}x_{3ref}+\frac{B_{eq}}{J_{eq}}{\dot{x}}_{3ref}+{\ddot{x}}_{3ref}\right)-\left(\frac{T_{r}}{J_{eq}N}+d_{1}^{2}e_{2}\right)-d_{2}e_{3}\\ =f_{1}(x)-d_{2}e_{3} \end{array}$$ (58)

Taking the derivative of (49), we get (59).


$${\dot{e}}_{4}={\dot{x}}_{5}-{\dot{\lambda }}_{2}$$ (59)

The second virtual control variable is selected according to condition (60), where *K*_1_ is a proportional coefficient between the fifth state variable (*x*_5_) and the reference variable (*x*_3ref_).


$$\lambda_{2}=K_{1}x_{3ref}$$ (60)

The Eq (61) is obtained by substituting Eqs (25) and (60) into (59).


$${\dot{e}}_{4}=-\frac{K_{t}}{L_{m}}x_{4}-\frac{K_{1}R_{m}}{L_{m}}x_{3ref}-K_{1}{\dot{x}}_{3ref}+\frac{1}{L_{m}}u_{2}(t)-\frac{R_{m}}{L_{m}}e_{4}=f_{2}(x)+\frac{1}{L_{m}}u_{2}(t)-\frac{R_{m}}{L_{m}}e_{4}$$ (61))

The second control signal, *u*_2_(*t*), is selected according to (62). When *e*_4_ is zero, calculating the value of *u*_2_(*t*) becomes extremely difficult. This value can be approximated based on a method proposed in ref [55].


$$u_{2}(t)=-L_{m}[\frac{e_{3}(e_{2}+f_{1}(x))}{e_{4}}+f_{2}(x)]$$ (62)

**Fig 4:**
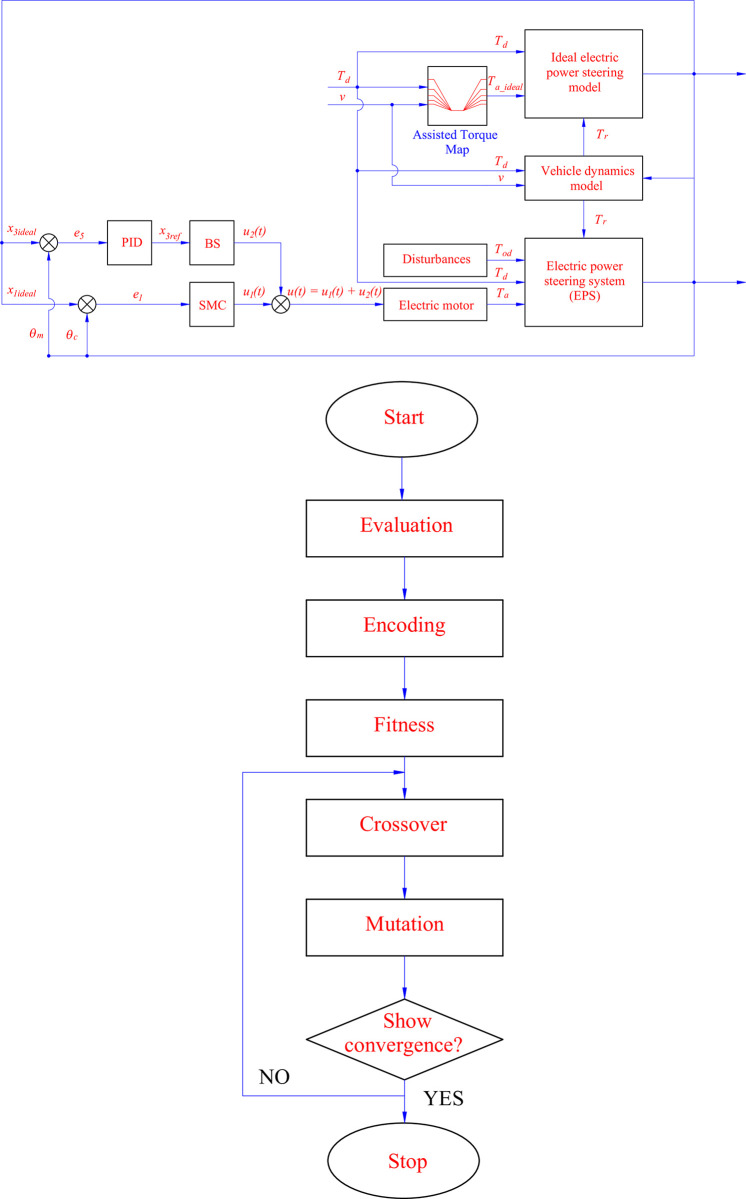
Control system diagram. a) Control scheme, b) Optimal algorithm workflow.

The diagram of the control system is illustrated in Fig 4A.

#### 2.2.1. Stability proofs

The first Lyapunov function is chosen according to (39), and the second Lyapunov function is chosen according to (63). These choices ensure that both functions are positive-definite ∀*x* ≠ 0. Combining (39) and (63), we obtain the equivalent Lyapunov function of the system, which is described in Eq (64).


$$V_{2}(x)=\frac{1}{2}(e_{2}^{2}+e_{3}^{2}+e_{4}^{2})>0$$ (63)

$$V(x)=V_{1}(x)+V_{2}(x)=\frac{1}{2}\sigma^{2}+\frac{1}{2}(e_{2}^{2}+e_{3}^{2}+e_{4}^{2})>0$$ (64)

Taking the derivative of (64), we get (65).


$$\dot{V}(x)={\dot{V}}_{1}(x)+{\dot{V}}_{2}(x)=\sigma \dot{\sigma }+e_{2}{\dot{e}}_{2}+e_{3}{\dot{e}}_{3}+e_{4}{\dot{e}}_{4}$$ (65)

Substituting Eqs (43), (44), (52), (58), and (61) into (65), we get (66).


$$\begin{array}{l} \dot{V}(x)=\sigma \left\{x_{1ref}^{\left(5\right)}-[f(x)+f(T_{d})+f(T_{r})+f(u_{1}(t))]+\sum_{i=1}^{4}k_{i}e_{1}^{\left(5-i\right)}\right\}\\ +(-d_{1}e_{2}^{2}-d_{2}e_{3}^{2}-d_{3}e_{4}^{2})+(e_{2}e_{3}+e_{3}f_{1}(x)+e_{4}f_{2}(x)+\frac{e_{4}}{L_{m}}u_{2}(t)) \end{array}$$ (66)

The coefficient *d*_3_ in Eq (66) is defined according to (67).


$$d_{3}=\frac{R_{m}}{L_{m}}$$ (67)

According to (68), the derivative of the equivalent Lyapunov function is negative definite ∀*x* ≠ 0 if and only if the first and second control signals are chosen according to (45) and (62), respectively. This is only true when the coefficient *d*_1_ satisfies condition (69). Combining (64) and (68), the system is considered stable.


$$\dot{V}(x)=-\sigma Ksat(\sigma )-d_{1}e_{2}^{2}-d_{2}e_{3}^{2}-d_{3}e_{4}^{2}<0$$ (68)

$$0<d_{1}<\frac{B_{eq}}{J_{eq}}$$ (69)

The input signal of the backstepping controller is calibrated through the PID algorithm. This relationship is described by Eq (70), where *e*_5_ is the third virtual error of the system and is calculated according to (71). The parameters of the PID controller are optimally calculated by a genetic algorithm. The algorithmic workflow is depicted in Fig 4B with seven stages. The structure of the algorithm should be referred to in [56].


$$x_{3ref}=k_{p}e_{5}+k_{i}\int e_{5}dt+k_{d}{\dot{e}}_{5}$$ (70)

$$e_{5}=x_{3ideal}-x_{3}$$ (71)

Once the controller design process is complete, calculations and simulations should be performed.

## 3. Result and discussion

### 3.1. Simulation conditions

**Fig 5:**
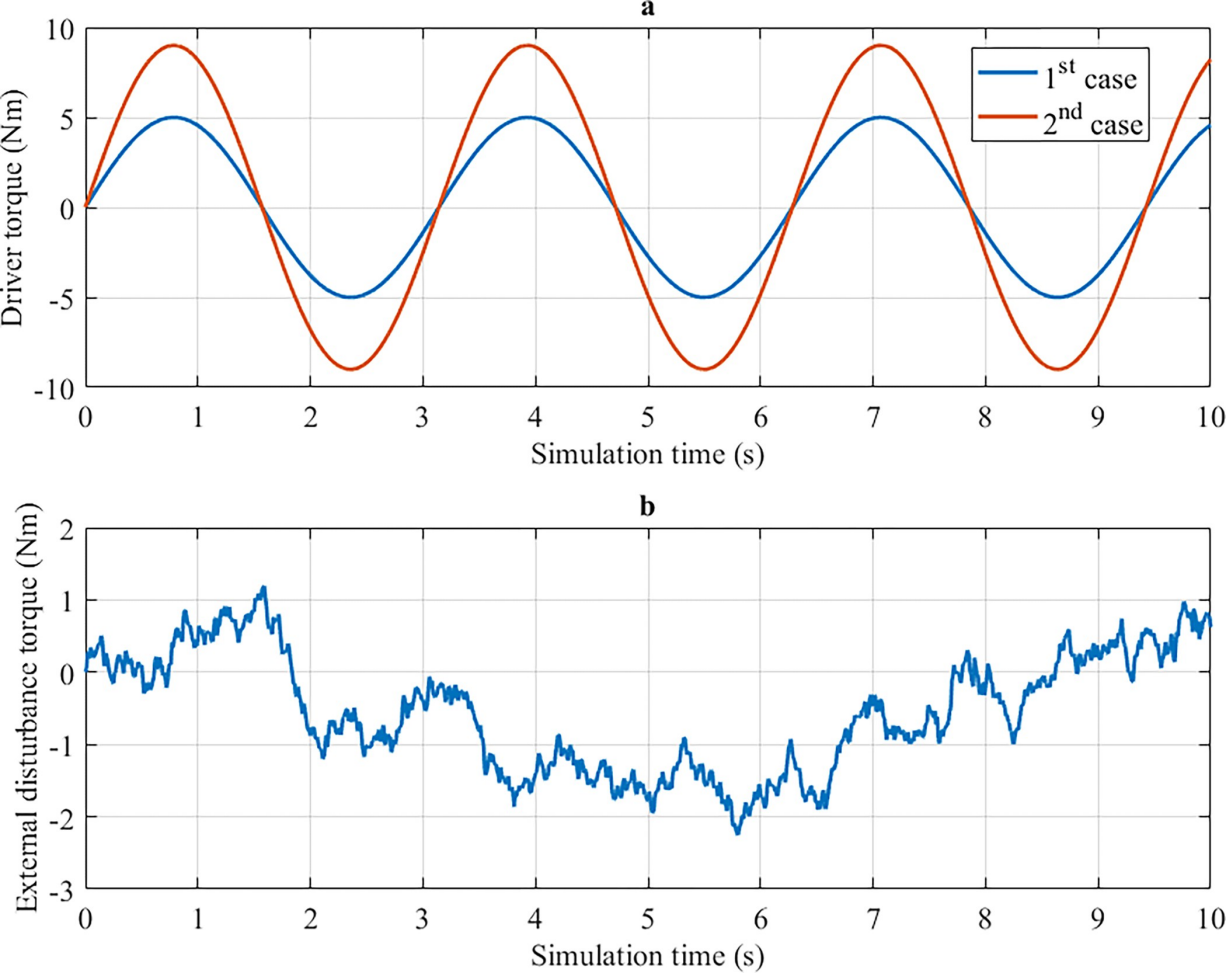
Simulation conditions. a) Driver torque, b) External disturbances torque.

A numerical simulation is performed to evaluate the results of the control algorithm, which is designed in this article. This work mentions two cases corresponding to two types of input (driver torque). These two simulation cases are illustrated in Fig 5A, while external disturbances are depicted in Fig 5B. External disturbances include road surface excitation, crosswinds, and environmental conditions. A white noise source generates the value used in the simulation.

The technical parameters of the EPS system and the vehicle dynamics model are shown in Table 1. The results of simulation include steering column angle, steering column speed, steering motor angle, steering motor speed, motor current, and assisted torque. These results are compared under five scenarios: Failure (the EPS system fails, and the user has to try hard to steer), PID, BS, BSSMCPID, and Reference. The outputs are obtained corresponding to three different speed values: *v*_1_ = 20 km/h (low speed), *v*_2_ = 50 km/h (medium speed), and *v*_3_ = 85 km/h (high speed).

**Table 1:** Simulation specifications.

Symbol	Value	Unit	Symbol	Value	Unit
*J*_*c*_	0.07	kgm^2^	*r*_*p*_	0.007	m
*B*_*c*_	0.072	Nms/rad	*l*_1_	1.20	m
*K*_*c*_	110	Nm/rad	*l*_2_	1.81	m
*L*_*m*_	0.005	H	*J*_*ψ*_	4350	kgm^2^
*R*_*m*_	0.45	Ω	*C*_*α*1_	45500	N/rad
*K*_*t*_	0.06	Nm/A	*C*_*α*2_	45500	N/rad
*N*	17.5		*l*_*n*_	0.032	m
*J*_*m*_	0.0005	kgm^2^	*M*	1700	kg
*B*_*m*_	0.0043	Nms/rad	*γ*_*p*_	11	°
*M*_*r*_	30	kg	*γ*_*c*_	4	°
*K*_*r*_	43200	N/mrad	*l*_*n*_	0.31	m
*B*_*r*_	3955	Ns/m	*l*_*c*_	0.032	m

### 3.2. Simulation results

This section presents the simulation results obtained from the two cases mentioned above.

#### 3.2.1. The first case

**Fig 6:**
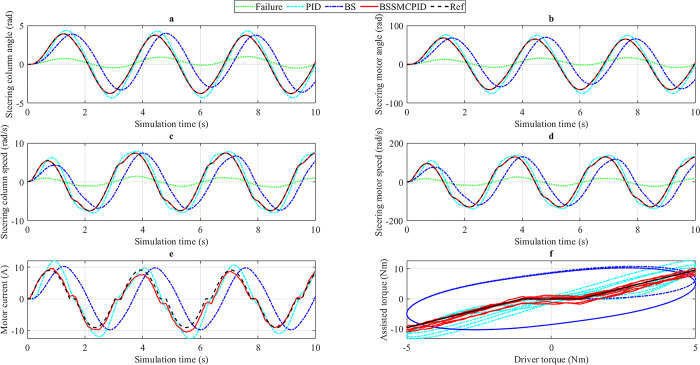
Simulation result (1^st^ case, *v*_1_). a) Steering column angle, b) Steering motor angle, c) Steering column speed, d) Steering motor speed, e) Motor current, f) Assisted torque.

*3*.*2*.*1*.*1*. *v*_*1*_ *= 20 km/h*. In the first case, a sine periodic signal is used as input (driver torque). The amplitude of this driver torque is 5 Nm, and its frequency is 2 rad/s (Fig 5A). The change in output variables when the car steers at low speed (*v*_1_ = 20 km/h) is illustrated in Fig 6.

The change in steering column angle (*x*_1_) is depicted in Fig 6A. According to numerical simulation results, the maximum reference value of the steering column angle is 3.93 rad, and the RMS reference value is 2.43 rad. These values are obtained from the ideal EPS system model. When the electric power steering system has a problem (Failure), these values decrease sharply to 1.00 rad and 0.51 rad, respectively. The PID controller maintains a stable state for the system under this condition. As a result, the peak value of the steering column angle (controlled by the PID controller) is 4.38 rad, while its RMS value is 2.76 rad, which is 0.45 rad and 0.33 rad higher than the desired value. A slight phase difference also occurs when using this linear controller. The error in the results can be slightly reduced by replacing the PID controller with the single BS controller. According to the research findings, the value of the steering column angle (obtained from the BS scenario) is 3.99 rad (peak value) and 2.46 rad (RMS value), respectively. Compared to PID, the difference between BS and Reference is smaller, only 0.06 rad and 0.03 rad. However, the phase delay in this scenario is more significant than in the PID scenario. The system error is almost completely eliminated once the BSSMCPID integrated controller is used. The calculation results show that the error between the BSSMCPID and Reference scenarios is approximately zero (the results have been rounded). The system response is excellent, and the phase difference phenomenon does not occur in this scenario.

Fig 6C illustrates the value of steering column speed (*x*_2_) when steering at low speed. The influence of external disturbance torque is shown in some oscillation locations on the graphs. The output signal obtained from the BS scenario is stable and robust. This shows that nonlinear control algorithms (such as BS) can provide high system stability even when subjected to strong external influences. However, the phase difference phenomenon still exists when only the single BS algorithm is applied. The peak and RMS values of the steering column speed obtained from this scenario are 7.44 rad and 4.36 rad, respectively, 0.01 rad and 0.54 rad lower than the desired values. Compared with BS, the results obtained from the PID controller are much more significant. These values amount to 8.03 rad for the peak value and 5.52 rad for the RMS value. There is a slight phase difference between the PID and Reference scenarios, while the phase difference between BS and Reference is more considerable. Looking at Fig 6C more closely, one can see that the output signal (*x*_2_) obtained from the BSSMCPID scenario always closely follows the reference signal. According to the numerical simulation results, their error is zero after the results have been rounded. The system remains stable once controlled by the BSSMCPID algorithm designed for this work, even when subjected to strong external disturbances.

The first controlled object is the steering column angle (*x*_1_), while the second is the steering motor angle (*x*_3_). The change of the second controlled object over time is illustrated in Fig 6B. The change trend of *x*_3_ is similar to *x*_1_, but its value is much larger. At low speed (*v*_1_ = 20 km/h), the electric motor works harder, causing the value of the steering motor angle to increase to 76.12 rad (peak value) and 47.95 rad (RMS value). These values are achieved when the system is controlled by the simple PID controller. Compared to the expected value, these numbers are 11.74% and 13.57% higher, respectively. Under the control of the BS controller, the system error is reduced to 2.66% (peak value) and 1.92% (RMS value). Compared with PID, the performance of the BS controller is preferable. However, the phase difference of BS is higher than PID, causing the system delay to increase. According to the numerical simulation results illustrated in Table 2, the maximum and RMS errors between the BSSMCPID and Reference scenarios are approximately zero. In addition, the phase difference in this scenario is almost nonexistent. The output signal always follows the reference signal, even when the system is strongly affected by external disturbances. A sharp decline is shown through the Failure scenario, causing the values to reduce to 17.20 rad and 8.77 rad.

**Table 2:** Simulation results (1^st^ case).

	Failure		PID		BS		BSSMCPID		Reference	
	Max	RMS	Max	RMS	Max	RMS	Max	RMS	Max	RMS
*v*_1_ = 20 km/h										
Steering column angle (rad)	1.00	0.51	4.38	2.76	3.99	2.46	3.93	2.43	3.93	2.43
Steering column speed (rad/s)	1.47	0.81	8.03	5.52	7.44	4.36	7.45	4.90	7.45	4.90
Steering motor angle (rad)	17.20	8.77	76.12	47.95	69.93	43.03	68.12	42.22	68.12	42.22
Steering motor speed (rad/s)	25.42	13.71	139.71	95.77	129.44	76.31	129.50	84.94	129.50	84.94
*v*_2_ = 50 km/h										
Steering column angle (rad)	0.90	0.47	2.85	1.71	3.22	1.85	2.55	1.53	2.55	1.53
Steering column speed (rad/s)	1.50	0.81	5.39	3.77	6.09	3.50	4.87	3.41	4.87	3.41
Steering motor angle (rad)	15.39	8.06	49.20	29.62	56.05	32.20	43.91	26.42	43.91	26.42
Steering motor speed (rad/s)	25.76	13.57	93.37	65.08	106.24	60.58	83.96	58.65	83.96	58.65
*v*_3_ = 85 km/h										
Steering column angle (rad)	0.90	0.54	2.05	0.87	2.90	1.61	1.87	0.64	1.87	0.64
Steering column speed (rad/s)	1.55	0.74	4.20	3.78	5.40	4.09	3.62	3.44	3.62	3.44
Steering motor angle (rad)	15.36	9.60	35.14	15.28	50.11	28.15	32.02	11.27	32.02	11.27
Steering motor speed (rad/s)	26.41	11.35	72.00	64.41	92.87	69.78	61.89	58.44	61.89	58.44

The change in steering motor speed (*x*_4_) is illustrated in Fig 6D. The error between the BSSMCPID and Reference scenarios is almost zero, while the error in the remaining scenarios is much larger. The values obtained from the PID scenario are more significant than the reference values: 7.88% (peak value) and 12.75% (RMS value), while the values obtained from the BS scenario are smaller than the reference values: 0.05% (peak value) and 10.16% (RMS value). A sharp decline occurs when the electric power steering system fails.

Power consumption performance is shown in Fig 6E. Motor current can be up to 12.58 A when the system is controlled by the traditional PID controller. This figure reduces to 10.22 A and 10.56 A for the BS and BSSMCPID controllers, while the desired value is 9.07 A. The RMS power consumption of the PID and BS scenarios is 6.82 A and 6.33 A, respectively, 20.92% and 12.23% higher than the desired value. In contrast, the difference between the BSSMCPID and Reference scenarios is only 2.48%. In general, energy efficiency is best when the system is controlled by the integrated nonlinear algorithm designed in this work.

Fig 6F depicts the relationship between driver torque (input) and assisted torque (output). The value of assisted torque obtained from the BSSMCPID scenario closely follows the Reference value with negligible error. According to the ideal assisted power steering curve (Fig 2), the value of assisted torque (*T*_*a*_) will increase linearly as |*T*_*d*_| > |*T*_*dmin*_| and *T*_*a*_ = 0 when |*T*_*d*_| < |*T*_*dmin*_|. The errors in the remaining two scenarios are more significant than in the BSSMCPID scenario. This proves that the performance of the BSSMCPID controller is much superior to that of single controllers.

*3*.*2*.*1*.*2*. *v*_*2*_ *= 50 km/h*. In this condition, the car’s speed increases from *v*_1_ = 20 km/h to *v*_2_ = 50 km/h. This aims to investigate the system’s assisted power efficiency at different speeds.

**Fig 7:**
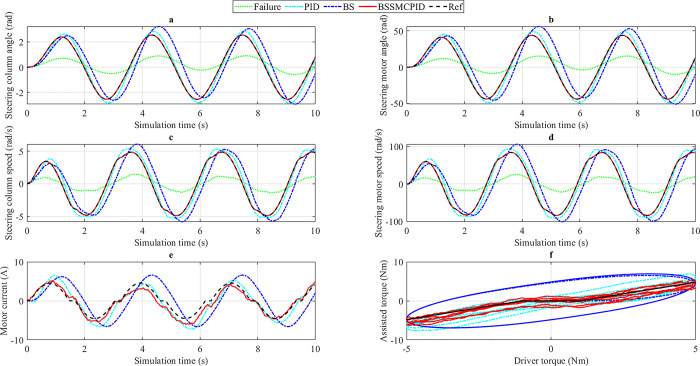
Simulation result (1^st^ case, *v*_2_). a) Steering column angle, b) Steering motor angle, c) Steering column speed, d) Steering motor speed, e) Motor current, f) Assisted torque.

The change in the steering column angle is clearly illustrated in Fig 7A. The ideal values of *x*_1_ are 2.55 rad (the maximum value) and 1.53 rad (the RMS value), which are lower than the *v*_1_ condition. When the system has the problem (Failure), the change in output results cannot meet the system’s requirements. A significant improvement is shown through using the PID controller for the electric power steering system. Simulation results show that the peak value of the steering column angle increases to 2.85 rad, 0.30 rad higher than the desired value, while its RMS value is 1.71 rad, 0.18 rad higher than the reference value. Under *v*_2_ conditions, the performance of the BS controller is worse than that of the PID. This is shown through the peak value and RMS value of the steering column angle (3.22 rad and 1.85 rad). The error between the BS and Reference scenarios is more significant than between the PID and Reference scenarios. Besides, the problem of phase difference still exists when we apply the backstepping technique to control the EPS system, while the phase difference of the PID technique is relatively tiny. Once the electric power steering system is controlled by the BSSMCPID algorithm, which is designed in this article, the error between the actual output signal and the desired signal is negligible. These values are assumed to be zero after the results have been rounded. The phase difference phenomenon does not occur when applying nonlinear integrated control techniques to the system.

Information related to steering motor angle is illustrated in Fig 7B. According to this description, a decline is clearly seen when the EPS system fails. The values obtained from this scenario are only about one-third of the desired values. The user needs to put more effort into controlling the direction of the car’s movement. A significant improvement in results is achieved when the system is controlled using the PID algorithm. The peak value of the steering motor angle is increased to 49.20 rad, while its RMS value is 29.62 rad, 12.05% and 12.11% higher than the Reference value. If the PID controller is replaced by the single BS controller, the maximum error of the system can be increased, while the RMS error can be reduced. The simulation results show that the systematic error of the BS scenario is 26.54% and 3.29%, in the order mentioned above. In this condition, the phase difference phenomenon still occurs strongly when applying the backstepping technique to the electric power steering system. The problems related to systematic errors and phase differences can be completely solved by applying the nonlinear integrated control technique called BSSMCPID. The error between the BSSMCPID and Reference scenarios is approximately zero, while the phase difference is almost non-existent. The system response is good even when subjected to external disturbances.

The variation of steering column speed and steering motor speed is illustrated in Fig 7C and 7D, respectively. The changing trends of *x*_2_ and *x*_4_ are similar. The most significant difference is their value. Generally, these values decrease as speed increases (driver torque is unchanged). The most significant peak error belongs to the BS scenario, while the most significant RMS error belongs to the PID scenario. However, the error between the BSSMCPID and Reference scenarios is considered zero once the results are rounded according to the set conditions.

Fig 7E provides information about the power consumption of different controllers. According to this data, the RMS power consumption of the PID and BS scenarios is significantly larger than the Reference, while the error of the BSSMCPID scenario is only about 6.99%. According to the last subplot (Fig 7F), assisted torque decreases as velocity increases. The error between the BSSMCPID and Reference scenarios is the smallest, while the errors in the other scenarios are significantly larger.

**Fig 8:**
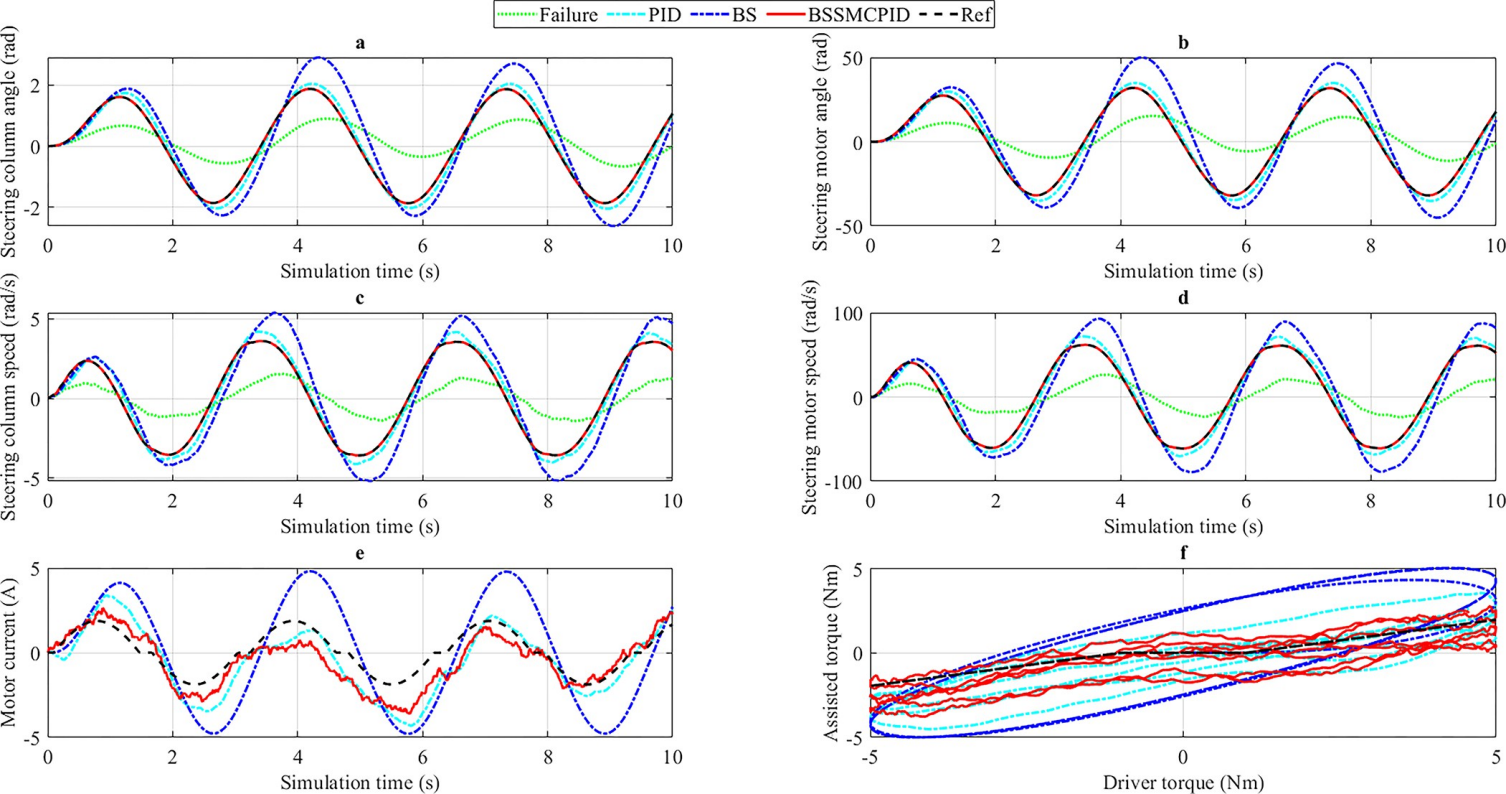
Simulation result (1^st^ case, *v*_3_). a) Steering column angle, b) Steering motor angle, c) Steering column speed, d) Steering motor speed, e) Motor current, f) Assisted torque.

*3*.*2*.*1*.*3*. *v*_*3*_ *= 85 km/h*. The power steering characteristic curve (Fig 2) shows that power steering performance will decrease significantly when steering at high speed. Therefore, it is necessary to investigate the system’s stability at speed *v*_3_ = 85 km/h. Fig 8 provides information about the output results when steering at high speed.

The changes in the controlled variables *x*_1_ and *x*_3_ are depicted in Fig 8A and 8B, respectively. Looking at Fig 8A more closely, we can see that the error of the BS scenario is larger than the PID. The data in Table 2 show that the maximum value and RMS value of the steering column angle achieved from the PID scenario are 2.05 rad and 0.87 rad, respectively, while the figures belonging to the BS scenario are 2.90 rad and 1.61 rad, respectively. These results are much higher than the reference values (1.87 rad and 0.64 rad). The system always reaches a steady state with approximately zero error if and only if the system is controlled by the BSSMCPID algorithm, which was designed in this article. Additionally, phase differences still occur for the PID scenario (slightly) and the BS scenario (strongly), while this does not happen for the BSSMCPID scenario. For the second controlled variable (*x*_3_), the errors between the PID and Reference scenarios are 9.74% (peak value) and 35.58% (RMS value), respectively (Fig 8B). These figures increase to 56.56% and 149.78% when the BS controller replaces the PID controller. This shows that the performance of both controllers drops sharply when steering at high speed, especially for the single nonlinear controller (BS). System performance is always guaranteed when the BSSMCPID algorithm controls the EPS system. As a result, the systematic error of the signals is approximately zero (after rounding).

As velocity increases, output values tend to decrease. The decline in steering column angle and steering motor angle is more significant than that of steering column speed (Fig 8C) and steering motor speed (Fig 8D). Furthermore, the peak values decrease more sharply than the RMS values. This reduction is valid for motor current (Fig 8E) and assisted torque (Fig 8F). According to the results of the last subplot, the assisted torque generated by the electric motor (after being amplified by a pair of gears) is relatively small. This perfectly agrees with the ideal assisted power steering curve (Fig 2). The simulation results in the first case are listed in Table 2.

The driver torque in the first case is insignificant. Therefore, it is necessary to use a more considerable value (|*T*_*d*_| > |*T*_*dmax*_|) to investigate the system’s quality in saturation state. This work will be done in the following subsection.

#### 3.2.2. The second case

In the second case, the amplitude of the input signal increases from 5 Nm to 9 Nm, exceeding its limit value (Fig 5A). The investigated work in this case is similar to the first case.

**Fig 9:**
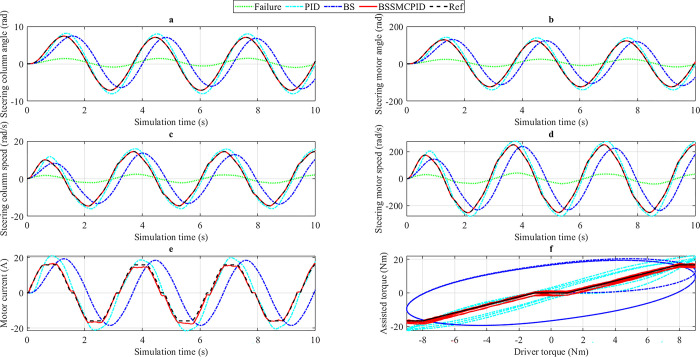
Simulation result (2^nd^ case, *v*_1_). a) Steering column angle, b) Steering motor angle, c) Steering column speed, d) Steering motor speed, e) Motor current, f) Assisted torque.

*3*.*2*.*2*.*1*. *v*_*1*_ *= 20 km/h*. The assisted power performance is most robust when the vehicle steers at low speed (*v*_1_ = 20 km/h). This is shown through the subplots in Fig 9. Fig 9A provides information about the change in steering column angle over time. The reference value is determined by 7.42 rad for the peak value and 5.93 rad for the RMS value. This value is 1.89 times and 2.44 times larger (in order) than the first case. If the steering system fails, the output value will decrease sharply to 1.49 rad and 1.36 rad. The user must put in more effort to achieve the ideal state (Reference). This causes a feeling of fatigue and a loss of comfort when used. The value of the steering column angle is well controlled when the EPS system is controlled by controllers, which this article discusses. The achieved values for the traditional PID controller are 8.18 rad and 7.20 rad, respectively, 10.24% and 21.42% higher than the desired threshold. This is a huge error which negatively affects the quality of the system. This can be improved slightly by using the single BS nonlinear controller instead of the PID. As a result, the systematic error is reduced to 0.54% and 13.66%, respectively. The error of the peak value is tiny, but the error of the RMS value is quite large. Besides, the phase difference phenomenon still occurs strongly when applying both algorithms. To solve the above problems completely, the BSSMCPID nonlinear integrated controller is proposed to be used. Simulation results show that the error between them is approximately zero (after the results have been rounded), and the phase difference problem does not exist.

The changing trend of the steering motor angle (Fig 9B) is similar to the steering column angle (Fig 9A). According to the system’s equations, these two state variables are closely related to each other. The desired values obtained from the ideal model (Reference) are 128.37 rad (peak value) and 103.47 rad (RMS value). If the system is controlled by the simple PID algorithm, the system error is 13.58 rad and 22.07 rad, respectively. These values decrease to 1.53 rad and 14.19 rad once the PID controller is replaced by the BS controller. According to the research findings, the error of the output value obtained from the BSSMCPID controller is only 0.01 rad, much smaller than in other scenarios. This helps ensure the accuracy and performance of the system.

As driver torque increases, the values of steering column speed (Fig 9C) and steering motor speed (Fig 9D) also increase, although the car speed does not change (*v*_1_ = 20 km/h). The simulation results show that the error of the PID scenario is more significant than that of the BS scenario, while the error of the BSSMCPID scenario is only about 0.01 rad/s. The output signal obtained from the BS scenario is highly stable against the influence of external disturbances, while the signal obtained from the PID is more strongly affected.

The motor current in the second case is larger than the first case (Fig 9E). The peak value of the current can be up to 21.54 A when applying the traditional PID algorithm, while the BS nonlinear controller can only generate a current with a peak amplitude of 19.31 A. These values are more significant than the desired value (15.88 A). When applying the nonlinear integrated algorithm proposed in this work, the peak value of the current is only 17.71 A, while its RMS error is only about 7.91%.

The increased current value causes the assisted torque to increase. The simulation results in Fig 9F show that assisted torque (*T*_*a*_) increases linearly as |*T*_*dmin*_| < |*T*_*d*_| < |*T*_*dmax*_| and *T*_*a*_ = *T*_*amax*_ (saturation state) when |*T*_*d*_| > |*T*_*dmax*_|. The slightest error belongs to the BSSMCPID scenario, while the errors in the other two scenarios are much more significant.

*3*.*2*.*2*.*2*. *v*_*2*_ *= 50 km/h*. When the speed increases from *v*_1_ = 20 km/h to *v*_2_ = 50 km/h, the output values will tend to decrease. This is a reasonable prediction based on the simulation results of the first case.

**Fig 10:**
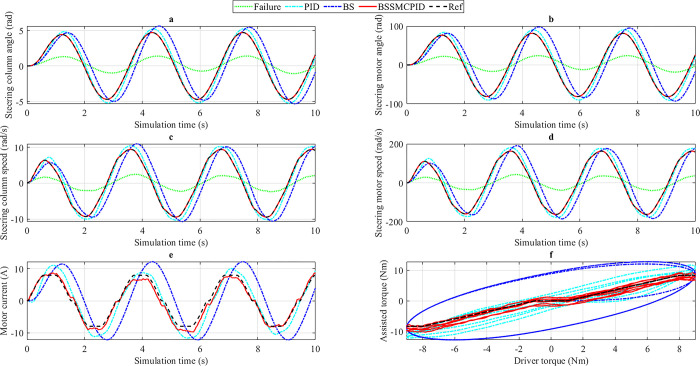
Simulation result (2^nd^ case, *v*_2_). a) Steering column angle, b) Steering motor angle, c) Steering column speed, d) Steering motor speed, e) Motor current, f) Assisted torque.

According to the results illustrated in Fig 10A, the errors of the steering column angle’s peak value and RMS value are 11.23% and 23.74%, respectively. If the single BS controller is used to replace the PID, this error even increases to 19.28% and 43.03%. The quality and responsiveness of the system will be seriously degraded if the error increases too much. This article proposes using the nonlinear integrated controller called BSSMCPID to maintain system stability. Simulation results show that the error of the BSSMCPID scenario compared to the Reference is approximately zero. In addition, the phase difference is also eliminated. For the steering motor angle, the error obtained from the nonlinear integrated controller designed in this work is only 0.01 rad, much smaller than that of other controllers (Fig 10B).

The BSSMCPID controller’s results are approximately ideal (Reference) with negligible errors. This is true for uncontrolled state variables, including steering column speed (*x*_2_) and steering motor speed (*x*_4_). According to the research findings, the error of *x*_2_ is approximately zero (Fig 10C), while the error of *x*_4_ does not exceed 0.01 rad/s (Fig 10D).

Unlike condition *v*_1_, the power consumption of the BS controller in condition *v*_2_ is more significant than PID. Their peak values are 12.21 A (BS) and 11.67 A (PID), much higher than the desired value (7.96 A). The error of the peak current and RMS values obtained from the BSSMCPID scenario is the smallest, only equal to 22.99% and 4.82% (Fig 10E). The changing trend of assisted torque in condition *v*_2_ is similar to *v*_1_, but its value is smaller (Fig 10F). The system reaches saturation state when |*T*_*d*_| > |*T*_*dmax*_|.

*3*.*2*.*2*.*3*. *v*_*3*_ *= 85 km/h*. In the final condition, the vehicle’s speed increases to *v*_3_ = 85 km/h. Once the speed increases, the output values will decrease sharply to increase the user’s steering feel and improve safety when steering at high speed.

**Fig 11:**
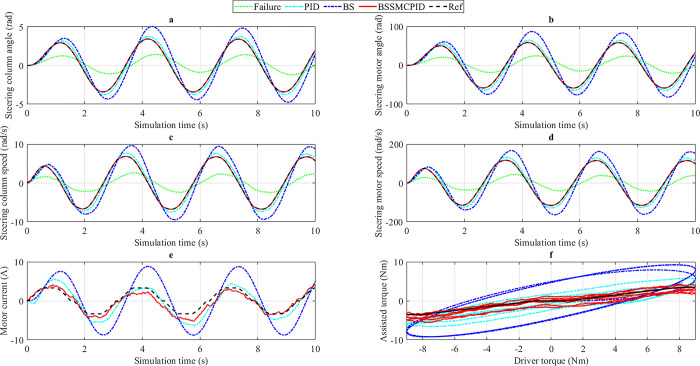
Simulation result (2^nd^ case, *v*_3_). a) Steering column angle, b) Steering motor angle, c) Steering column speed, d) Steering motor speed, e) Motor current, f) Assisted torque.

The simulation results under this condition are shown in Fig 11. In general, the changing trend of the output results is similar to the conditions previously investigated. The value of the outputs (state variables) always closely follows the desired value if and only if the system is controlled by the BSSMCPID algorithm, which is proposed in this work. These errors are minimal and can even be considered zero after the results have been rounded. The errors in the remaining two scenarios (PID and BS) are more significant, and the phase difference phenomenon still exists. The amount of power consumption in the BSSMCPID scenario is the smallest, close to the desired value. The decline of assisted torque when steering at high speed is depicted in the last subplot in Fig 11. In general, the error of the BSSMCPID controller is insignificant compared to the remaining controllers.

The simulation results in the second case are listed in Table 3.

**Table 3:** Simulation results (2^nd^ case).

	Failure		PID		BS		BSSMCPID		Reference	
	Max	RMS	Max	RMS	Max	RMS	Max	RMS	Max	RMS
*v*_1_ = 20 km/h										
Steering column angle (rad)	1.49	1.36	8.18	7.20	7.46	6.74	7.42	5.93	7.42	5.93
Steering column speed (rad/s)	2.41	0.73	16.05	7.16	13.65	4.03	14.56	8.10	14.56	8.10
Steering motor angle (rad)	25.49	23.68	142.31	125.54	130.26	117.66	128.72	103.46	128.73	103.47
Steering motor speed (rad/s)	41.61	10.91	278.60	122.86	237.87	72.03	252.55	136.59	252.54	136.58
*v*_2_ = 50 km/h										
Steering column angle (rad)	1.40	0.90	5.25	4.17	5.63	4.82	4.72	3.37	4.72	3.37
Steering column speed (rad/s)	2.49	0.83	10.46	6.45	10.95	2.70	9.43	6.81	9.43	6.81
Steering motor angle (rad)	23.89	15.72	90.80	72.66	97.96	84.08	81.42	58.81	81.43	58.82
Steering motor speed (rad/s)	42.51	12.42	180.55	110.65	191.03	45.54	162.59	114.79	162.60	114.79
*v*_3_ = 85 km/h										
Steering column angle (rad)	1.43	0.82	3.77	2.37	5.05	2.91	3.43	2.18	3.43	2.18
Steering column speed (rad/s)	2.61	1.63	7.69	5.17	9.70	6.26	6.85	4.71	6.85	4.71
Steering motor angle (rad)	24.41	13.76	64.62	40.69	87.10	50.10	58.70	37.28	58.70	37.28
Steering motor speed (rad/s)	44.33	27.33	131.91	88.59	166.60	107.94	117.06	80.57	117.06	80.57

From the above results, some conclusions are drawn as follows:

+ If the velocity does not change while the driver torque increases, the variation of the output values will increase. If the driver torque does not change while the speed increases, the variation of the output values will decrease. This is true for all scenarios except (Failure).

+ The value of assisted torque increases linearly when |*T*_*d*_| > |*T*_*dmin*_|, and it reaches saturation when |*T*_*d*_| > |*T*_*dmax*_|.

+ The BS algorithm provides robust stability even when the system is subjected to external disturbances. However, the phase difference of the output signal is quite large when the system is controlled by this algorithm.

+ In the first case (driver torque is small), the BS algorithm performs superior to PID when the vehicle steers at low and medium speeds (*v*_1_ and *v*_2_). On the contrary, the performance of the BS algorithm seriously degrades when the vehicle steers at high speed (*v*_3_). In the second case (driver torque is large), the PID algorithm provides slightly superior performance compared to the single BS algorithm.

+ When applying the nonlinear integrated algorithm BSSMCPID, the system error is significantly reduced compared to other algorithms. In most simulation conditions, the value of the RMS error is approximately zero (results have been rounded). The output signal from this scenario always closely follows the reference signal with negligible error. In addition, the phase difference phenomenon does not occur when applying this technique to control the EPS system.

## 4. Conclusion

The steering process will become more convenient and comfortable when the EPS system is installed on a car. A nonlinear integrated control algorithm is established in this article to apply to the EPS system. This novel algorithm combines two nonlinear control techniques, BS and SMC, with the input regulated through PID technology. A numerical simulation process is performed to evaluate the quality of the proposed algorithm.

The simulation result show that the output values (state variables) tend to closely follow the reference value with extremely small errors if and only if the system is controlled by the BSSMCPID method. Under some conditions, the systematic error is considered approximately zero, and the phase difference phenomenon is completely eliminated. The system response is excellent even when subjected to external disturbance torque. Additionally, the system’s power consumption is also strongly reduced when the system is controlled by the nonlinear integrated algorithm proposed in this work, compared to other algorithms such as BS or PID.

The new integrated algorithm provides outstanding performance in ensuring system stability and robustness. However, two problems still exist and cannot be resolved. Firstly, the actual motor current signal does not follow the reference value. Secondly, the road reaction torque (*T*_*r*_) has only been calculated based on the linear single-track model instead of a spatial nonlinear model. These two issues should be resolved in future studies. In addition, conducting future experiments is necessary to determine the performance of the proposed algorithm accurately.

## Abbreviation

ACO: Ant colony optimization
ADRC: Active disturbance rejection control
BCGA: Binary–code genetic algorithm
BLDC: Brushless direct current
BPNN: Back propagation neural network
BS: Backstepping
ECM: Electronic control module
EHPS: Electrohydraulic power steering
EPS: Electric power steering
HPS: Hydraulic power steering
LPV: Linear parameter–varying rad
LQR: Linear quadratic regulator rad
MPC: Model predictive control rad
MSE: Mean square error rad
PID: Proportional–integral–derivative rad
PSO: Particle swarm optimization rad
RMS: Root mean square rad
SMC: Sliding mode control rad
*δ*: Steering angle rad
*α*: Slip angle rad
*β*: Heading angle rad
*ψ*: Yaw angle rad
*θ_c_*: Steering column angle rad
*θ_m_*: Steering motor angle rad
*γ_c_*: Caster angle rad
*γ_k_*: Kingpin angle rad
*li*: Center distance m
*Bc*: Steering column damping Nms/rad
*Beq*: Equivalent damping Nms/rad
*B_m_*: Motor damping Nms/rad
*B_r_*: Rack damping Ns/m
*i_m_*: Motor current A
*J_ψ_*: Yaw inertia moment kgm^2^
*J_c_*: Inertia moment of the column kgm^2^
*J_eq_*: Equivalent moment of inertia kgm^2^
*J_m_*: Inertia moment of the motor kgm^2^
*K_c_*: Steering column stiffness Nm/rad
*K_t_*: Motor torque coefficient Nm/A
*L_m_*: Electric motor inductance H
*l_n_*: Knuckle arm length m
*m*: Vehicle mass kg
*M_r_*: Rack mass kg
*N*: Gear ratio -
*R_m_*: Electric motor resistance Ω
*r_p_*: Pinion radius m
*T_a_*: Assisted torque Nm
*T_d_*: Driver torque Nm
*T_r_*: Road reaction torque Nm
*u(t)*: Control signal V
*v_x_*: Longitudinal velocity m/s
*v_y_*: Lateral velocity m/s
